# The Effects of Cost Containment and Price Policies on Pharmaceutical Expenditure in South Korea

**DOI:** 10.34172/ijhpm.2021.135

**Published:** 2021-09-21

**Authors:** Woohyeon Kim, Heejo Koo, Hye-Jae Lee, Euna Han

**Affiliations:** ^1^Korea Institute of Public Finance, Sejong, South Korea.; ^2^College of Pharmacy, Yonsei Institute of Pharmaceutical Research, Yonsei University, Seoul, South Korea.; ^3^College of Pharmacy, Woosuk University, Wanju, South Korea.

**Keywords:** Pharmaceutical Policy, Prescription Expenditure, Interrupted Time Series, Pharmaceutical Cost Containment

## Abstract

**Background:** Policy-makers have proposed and implemented various cost-containment policies for drug prices and quantities to regulate rising pharmaceutical spending. Our study focused on a major change in pricing policy and several incentive schemes for curbing pharmaceutical expenditure growth during the 2010s in Korea.

**Methods:** We constructed the longitudinal dataset from 2008-2017 for 12 904 clinics to track the prescriber behavior before and after the implemented policies. Applying an interrupted time series model, we analyzed changes in trends in overall monthly drug expenditure and antibiotic drug expenditure per prescription for outpatient claims diagnosed with three major diseases before and after the policies’ implementation.

**Results:** Significant price reductions and incentives for more efficient drug prescriptions resulted in an immediate decrease in monthly drug expenditures in clinics. However, we found attenuated effects over the long run. The top-spending clinics showed the highest rate of increase in drug costs.

**Conclusion:** Future policy interventions can maximize their effects by targeting high-spending providers.

## Background

**Key Messages**

** Implications for policy makers**

- The enforcement of price reduction had an immediate effect on lowering pharmaceutical expenditures.
- There was no reversal in the upward trend in the pharmaceutical expenditure as the total drug costs reached a higher rate than before introducing each policy.
- The policy effect on cost containment was most significant at high-volume clinics.
- More targeted policy measures for the higher spending groups are needed to enhance overall policy effectiveness.

** Implications for the public**

This study assessed the overall effects of four policies targeting pharmaceutical expenditure containment via direct price cuts or through changes in physicians’ prescription behaviors. Our results show that the increasing trend of the pharmaceutical expenditure per prescription was reduced immediately after price reduction. However, we did not find a policy maintenance effect as the upward cost trends reappeared. We also demonstrated that the policy effect on cost containment was largest for the high-volume clinics who showed the fastest growth in total expenditure during the study period. Our findings of a more significant increase in pharmaceutical expenditures among high-volume clinics imply that policy measures could target specifically high-spending clinics.

Pharmaceutical expenditures account for a significant portion of healthcare costs in Korea. In 2018, pharmaceutical spending was 14.9 billion dollars, 24.6% of total healthcare spending. A rapidly aging population and an increase in chronic disease prevalence have exacerbated the financial burden of medication costs.

 Pharmaceutical expenditure comprises drug prices and the drug quantity consumed,^1^ the two main policy levers on which the government focuses. Although every country uses different policies to contain pharmaceutical expenditures, we can narrow the policies into two main categories. The first is how a government controls drug pricing to supply necessary drugs under budget constraints. The second is how it incentivizes physicians and patients to utilize drugs in the most cost-efficient way. The evidence from one country may not be generalizable in a different societal context. Therefore, policy-makers and researchers continuously examine many countries’ policies to design the most appropriate combination.

 The period from the late-2000s to the mid-2010s in Korea is of particular interest, as this was when the country introduced a sequence of different policies regarding drug prices and physicians’ incentives. Pharmaceutical spending in Korea surged from 22% of total health expenditures in 1999 to 28.7% in 2006.^2^ In 2006, 32 OECD (Organisation for Economic Co-operation and Development) member countries spent an average of 19.1% of their healthcare budgets on drug consumption. The Korean government introduced several major incentive policies, including an Outpatient Prescription Incentive Program in October 2010, which rewarded financial incentives for physicians’ cost-reducing drug prescription behavior (eg, generic drug prescription). In addition, the Uniform Ceiling Prices for Generics Program in April 2012 enacted a markdown on list prices for a significant portion of the listed drugs. This markdown policy affected the prices of 6506 (47.1%) of the 13 814 drugs listed by Korean National Health Insurance in 2011, reducing prices by an average of 21%.^3^

 This study identifies the overall effects of a series of price and incentive policies on pharmaceutical expenditures implemented between 2010 and 2015 in Korea. The price policy directly affects the drug price and consequently reduces pharmaceutical spending, whereas the incentive policies induce changes in physicians’ prescribing behavior. We summarized all of the effects into the cost variable, which we mainly analyzed. There are several mechanisms through which these policies affect pharmaceutical spending. First, the price markdown directly reduces the cost per dose or pack, but it may indirectly increase demand due to relatively low prices. Second, prescription incentive programs may lead to changes in physicians’ prescribing behavior depending on how attractive the incentive is for the physician. Whether the cumulative effect of the different strategies leads to cost-saving prescription behaviors and ultimately reduces pharmaceutical expenditure is an empirical question.

 Several studies^4,5^ analyzed claims data in Korea to identify some of the policy effects on drug spending and physician behavior. However, the researchers only focused on short-term effects after policy implementation. In this paper, we analyzed National Health Insurance claims data from 2008 to 2017, allowing us to explore long-term policy impacts. As shown in research on European countries and Taiwan,^6-8^ drug price and incentive policies for physicians may have different long-term effects than a short-term result. Since prescription incentive programs may affect prescription behavior and drug consumption in the long run, it is essential to track relevant outcomes for a longer period.

## Methods

###  Policy Background

 In this study, we focused on four cost-containment policies implemented between 2010 and 2015 in Korea. The policies intended to alleviate pharmaceutical spending growth by lowering drug prices and changing prescription behaviors. More details on each policy follow.

####  Outpatient Prescription Incentive Program

 The National Health Insurance Service (NHIS) began to incentivize clinics to reduce pharmaceutical spending in October of 2010.^9^ When a clinic achieves a substantial drug expenditure saving on outpatient prescriptions, it becomes eligible for financial incentives. The incentive scheme depends on two components: how much a clinic prescribed in the current year compared to other clinics and how much a clinic prescribed in the current year compared to the previous year. First, NHIS calculates the total expected spending for the clinic based on its previous year’s prescriptions and compares actual and expected drug spending for each clinic. The base of the reward amount is the difference between the two, which represents how much money the clinic saved. Then, NHIS calculates a cost index that shows the clinic’s degree of relative drug spending compared to an average provider in the current year. An index for the clinic determines its incentive relative to the savings achieved by the clinic, ranging from 20-40%.^10^

####  Uniform Ceiling Prices for Generics

 Until April of 2012, the Korean government implemented a stepwise pricing policy for generic drugs depending on their listing order in the health insurance formulary.^11^ This stepwise pricing system was intended to rapidly introduce inexpensive generic drugs after patent expiration. However, it resulted in unexpectedly fierce competition among pharmaceutical companies to list their generic products early in the health insurance formulary, sometimes resulting in poor drug quality. In April of 2012, the Korean government abolished the stepwise pricing system. Instead, all generic drug price ceilings are now uniformly set at 53.55% of the original drug’s price before patent expiration.^12,13^

####  Pay-for-Performance Program 

 In 2001, the Health Insurance Review & Assessment Service (HIRA), responsible for reviewing medical claims, began monitored the prescription behavior of clinics and hospitals.^14^ In July of 2013, HIRA further began to evaluate drug utilization and incentivize more efficient pharmaceutical spending by adjusting each clinic’s reimbursement according to its prescription behavior.^15^ Prescription behaviors are assessed bi-annually by three indices: the antibiotic prescription rate for acute upper respiratory infections, the injection prescription rate, and the rate for polypharmacy prescriptions. The outpatient management fee (the main component of outpatient reimbursements) received an upward adjustment of ±1% for the relative performance of each index (total adjustments up to ±3%). This adjustment was on top of other incentive measures, such as the outpatient incentive scheme aforementioned. Starting in 2018, the adjustment rate of outpatients’ management fees went up to ±5%.^16^

####  Extended Incentive Program

 In July of 2014, the outpatient prescription incentive program was integrated into a broader plan, consisting of outpatient prescription incentives and low procurement price incentives for clinics, hospitals, and pharmacies.^17^ The latter incentivizes medical providers and pharmacies to purchase drugs at a lower price than the listed price ceiling. Therefore, the government expanded the incentive program to inpatient drug consumption in clinics and hospitals and pharmacy-dispensed medications to outpatients. Clinics, hospitals, or pharmacies can receive 10-30% savings for purchasing drugs lower than the listed price.^18^ In addition, the proportion of incentives to total savings from the outpatient prescription incentive program increased from 20%-40% to 10%-50% after this policy.

###  Data

**Figure 1:**
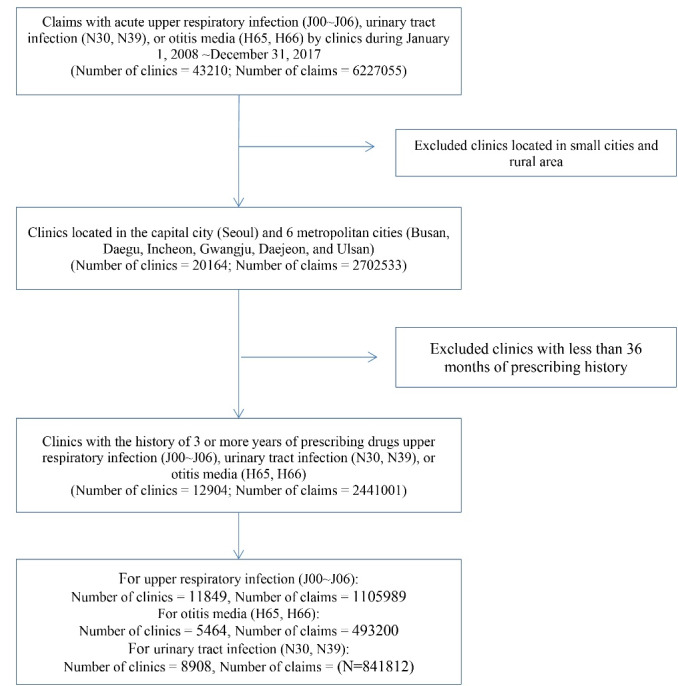
Flowchart of the Sample Selection.

 This study focused on three frequently-occurring outpatient conditions in Korea: acute upper respiratory infection (J00-J06), urinary tract infection (N30, N39), and otitis media (H65, H66). In 2018, the Korean National Health Insurance covered costs related to these conditions for 32.9 million patients, spending KRW 1718 billion (US$ 1.44 billion).^19^ We analyzed changes in J01 class (antibiotic) drug spending per prescription and total outpatient drug expenditure per prescription in response to the enacted policies. Korea is well-known for its high antibiotic use, which in 2018 was 29.8 defined daily dosage per 1000 inhabitants daily, the third-highest usage among OECD member countries.^20^ We focused on clinics in the capital city (Seoul) and six metropolitan cities (Busan, Daegu, Incheon, Gwangju, Daejeon, and Ulsan) in Korea, of which 43.8% of the Korean population resided as of February of 2019. We included only clinics with a history of three or more years of prescribing drugs for the target diseases in our sample (see Figure 1 for sample selection).

 We obtained aggregated monthly drug spending information for the three conditions and other relevant information for each clinic from the HIRA in Korea. We aggregated all of the information from insurance claims submitted by clinics to HIRA. We constructed a longitudinal dataset from 2008 to 2017 for 12 904 clinics to track prescribers’ behavior before and after policy implementation.

###  Variables and Analysis

 We adopted an interrupted time series model for analyzing the effects of relevant policies. Researchers widely use this model as it simultaneously identifies immediate policy effects and differences before and after these effects.^21^ The interrupted time series is appropriate for sequentially identifying policy effects.

 The four main policies we focused on are the Outpatient Prescription Incentive Program, the Uniform Ceiling Prices for Generics, the Pay-for-Performance Program, and the Extended Incentive Program. We generated a series of dummy indicators to represent each of those policies as *Policy1*, *Policy2*, *Policy3*, and *Policy4*. We coded each indicator as 1 for after the policy implementation period and 0 for before implementation. We divided the years from 2008 to 2017 into five different periods. The first period (2008.1-2010.9), *Time*0_t_specifies the time before any program implementation. The second period (2010.10-2012.3), *Time*1_t_ corresponds to the time following initiation of the Outpatient Prescription Incentive Program for clinics. The third period (2012.4-2013.7) *Time*2_t_ is the time after application of the Uniform Ceiling Prices for Generics. The fourth period (2013.7-2014.6) *Time*3_t_ indicates the time after implementation of the Pay-for-Performance Program. The final period (2014.7-2017.12) *Time*4_t_ is the time after the Extended Incentive Program (*Policy*4_t_) was in effect.

 The model for medical provider *i* at month *t* is as follows.

*Y*_it_ *= β*_0_ *+ β*_1_ *Policy1*_t_ *+ β*_2_ *Policy2*_t_ *+ β*_3_ *Policy3*_t_ *+ β*_4_ *Policy4*_t_ *+ α*_0_ *Time0*_t_ *+ α*_1_ *Time1*_t_ *+ α*_2_ *Time2*_t_ *+ α*_3_ *Time3*_t_ *+ α*_4_ *Time4*_t_ *+ γX*_t_ *+ λZ*_it_ *+ μ*_i_ *+ ϵ*_it_

 The model can identify both immediate effects of *Policy1, Policy2, Policy3, *and* Policy4* and their subsequent long-term effects via *Time*0_t _– *Time*4_t_. We were interested in the policy effects on the outcome variables *Y*_it_, the outpatient total drug spending per prescription, and outpatient antibiotic drug spending per prescription. The model is beneficial as it accounts for various factors that may confound the relationship between the outcomes and policy variables. We included other related policy changes during the sample period, *X*_t_ including introducing the drug utilization review (implemented in December 2010) and risk-sharing agreement (implemented in April 2014) as dummy indicators denoting time since the implementation of each policy. We also controlled for provider characteristics *Z*_it_ such as the clinic’s specialty (general practitioner, internal medicine, orthopedics, pediatrics, ophthalmology, ENT, dermatology, urology, family medicine, and others), years of operation (under 3, 3–5, 5–10, 10–20, over 20 years), and the clinic’s market share in a local market confirmed as a city/county/district as defined by the Herfindahl-Hirschman Index (HHI). To account for unobserved time-invariant factors, we included the provider fixed effect μ_i_.

 We were also interested in how individual providers react to policies and programs differently. In particular, we wanted to know how clinics that aggressively prescribe drugs for their patients, ie, clinics with high total drug spending or high antibiotic drug spending per patient visit, respond to the policies designed to change their prescription behavior. We fit 10%, 25%, 50%, 75%, and 90% quantile regressions to separately identify the effects of policies and programs on clinics with different prescription spending volumes.

## Results

Table 1 shows the summary characteristics of the study sample. General practitioners were the most dominant group among the study sample clinics (43% of 11 849 clinics for acute upper respiratory infections, 30% of 5464 clinics for otitis media, and 46% of 8908 clinics for urinary tract infections), followed by internal medicine specialists (19% for acute upper respiratory infections, 18% for otitis media, and 24% for urinary tract infections). The average practice duration was 11 to 12 years. The HHI was 2.4-6.5% on average, implying high market competitiveness (Table 1).

**Table 1:** Summary Statistics of the Primary Clinics Included in This Study

	**Acute Upper Respiratory Tract Infections, No. (%)**	**Otitis Media, ** **No. (%)**	**Urinary Tract Infections, No. (%)**
Monthly total drug expenditure per prescription (KRW, mean (SD))			
Time 0: Before the Outpatient Prescription Incentive Program (2008.1~2010.9)	6891.8 (12190.2)	7332.9 (16756.6)	12557.8 (13642.0)
Time 1: Between Policy 1 and Uniform Ceiling Prices for Generics (Policy 2) (2010.10~2012.3)	7525.3 (7158.2)	7719.9 (7700.6)	13715.9 (14522.1)
Time 2: Between Policy 2 and Pay-for-Performance Program (Policy 3) (2012.4~2013.6)	6796.8 (6396.8)	6617.2 (7467.1)	12103.2 (13150.5)
Time 3: Between Policy 3 and Extended Incentive Program (Policy 4) (2013.7-2014.6)	7097.6 (7253.7)	6782.4 (7404.0)	12523.3 (13706.0)
Time 4: After Policy 4 (2014.7~2017.12)	8069.1 (10194.8)	7381.2 (8143.4)	14019.5 (16962.2)
Monthly antibiotics expenditure per prescription (KRW, mean (SD))			
Time 0: Before the Outpatient Prescription Incentive Program (2008.1~2010.9)	2963.4 (2703.6)	3742.4 (16645.8)	4339.6 (3864.3)
Time 1: Between Policy 1 and Uniform Ceiling Prices for Generics (Policy 2) (2010.10~2012.3)	3465.9 (2877.1)	4154.8 (2665.9)	4947.5 (4015.7)
Time 2: Between Policy 2 and Pay-for-Performance Program (Policy 3) (2012.4~2013.6)	3004.1 (2299.1)	3412.6 (2350.9)	4197.6 (3194.3)
Time 3: Between Policy 3 and Extended Incentive Program (Policy 4) (2013.7-2014.6)	3111.6 (2632.0)	3498.7 (2243.4)	4182.6 (3163.9)
Time 4: After Policy 4 (2014.7~2017.12)	3351.5 (2650.8)	3822.3 (2583.1)	4220.4 (3163.4)
Specialty			
General practitioner	5138 (43.36)	1651 (30.22)	4091 (45.93)
Internal medicine	2239 (18.90)	963 (17.62)	2174 (24.41).
Orthopedics	902 (7.61)	40 (0.73)	364 (4.09)
Pediatrics	1227 (10.36)	1196 (21.89)	1073 (12.05)
Ophthalmology	158 (1.33)	2 (0.04)	2 (0.02)
Otorhinolaryngology	1252 (10.57)	1252 (22.91)	146 (1.60)
Dermatology	234 (1.97)	0 (0.00)	113 (1.26)
Urology	295 (2.59)	4 (0.07)	549 (6.16)
Family medicine	404 (3.41)	356 (6.52)	396 (4.45)
Duration of practice in years, Mean (SD)	11.646 (9.117)	11.302 (8.497)	12.326 (9.205)
HHI, Mean (SD)	0.024 (0.014)	0.065 (0.050)	0.031 (0.018)

Abbreviations: SD, standard deviation; HHI, Herfindahl-Hirschman Index. Note: During the study period (January 1, 2001 – December 31, 2017), US$ 1 was equivalent to KRW 1131.84, on average.

**Figure 2:**
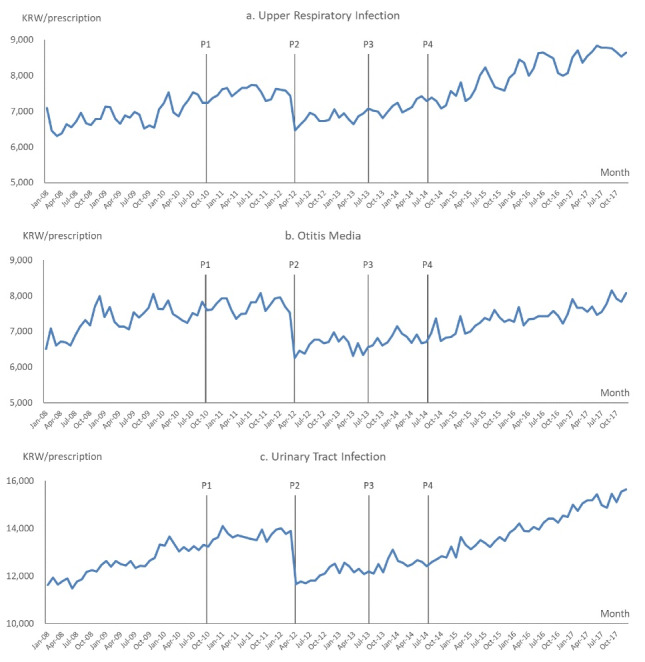
The Monthly Trend of Total Drug Expenditures Per Prescription Per Clinic. Note: P1. Outpatient incentive program (‘10–10’). P2. Uniform Ceiling Prices for Generics (‘12–4’). P3. Pay-for-performance program (‘13–7’). P4. Extended outpatient incentive program (‘14–7’). During the study period (January 1, 2001 – December 31, 2017), US$ 1 was equivalent to KRW 1131.84, on average.

Figure 2 displays the average monthly total drug expenditures per prescription by month and target disease by clinic. Monthly drug expenditures dropped after the Outpatient Prescription Incentive Program in October of 2010 (Policy 1) for otitis media and the universal drug-price cut by the Uniform Ceiling Prices for Generics in April of 2012 (Policy 2) for all three target diseases. We also observed drops in monthly drug expenditure for upper respiratory infections and urinary tract infections after the extended Outpatient Prescription Incentive Program in July of 2014 (Policy 4).

 We saw the largest drop after the Uniform Ceiling Prices for Generics (Policy 2). Monthly drug expenditures dropped by 12.9% for upper respiratory tract infections (from KRW 7429 to KRW 6465), 16.9% for otitis media (from KRW 7533 to KRW 6253), and 16.0% for urinary tract infections (from KRW 13902 to KRW 11671) compared to before the policy. (During the study period of January 1, 2001 – December 31, 2017, US$1 was equivalent to KRW 1131.84, on average). The decreases in monthly expenditures after the Extended Incentive Program (Policy 4) were 1.8% for upper respiratory infections and 1.4% for urinary tract infections. Minimal changes were reported after other policies were introduced.

 Also, the average per clinic monthly total drug expenditures per prescription by month rolled back to the previous levels or continued to increase. There was an immediate drop after the overall price cut by the Uniform Ceiling Prices for Generics Program (Policy 2) for all target diseases (Figure 2).

Table 2 shows the estimated results of the effects of pharmaceutical policies on pharmaceutical and antibiotic expenditures via an interrupted time series model. The immediate effects on the monthly total pharmaceutical expenditures were obvious for upper respiratory infections, as it dropped by an average of KRW 858 after the Uniform Ceiling Prices for Generics (Policy 2). However, the monthly total pharmaceutical expenditures increased approximately KRW 13, KRW 11, and KRW 14 per month in the three time segments (Time 2, Time 3, Time 4, respectively) after the Uniform Ceiling Prices for Generics Program (Policy 2). We estimated similar patterns for otitis media and urinary tract infections.

**Table 2:** Effects of Pharmaceutical Policies on Pharmaceutical and Antibiotic Expenditures

**Key Independent Variables and Intercepts**	**Regression Estimate (Standard Error)**					
	**Monthly Total Drug Expenditure Per Prescription ** ** (1000 KRW)**			**Monthly Antibiotics Expenditure Per Prescription ** ** (1000 KRW)**		
	**Upper Respiratory Infection**	**Otitis Media**	**Urinary Tract Infection**	**Upper Respiratory Infection**	**Otitis Media**	**Urinary Tract Infection**
Intercept	9.193 (0.337)^c^	7.071 (3.714)	20.845 (1.87)^c^	4.483 (0.218)^c^	6.353 (3.437)	6.387 (0.49)^c^
Time 0: Before the Outpatient Prescription Incentive Program (Policy 1) (2008.1~2010.9)	0.020 (0.004)^d^	0.025 (0.004)^d^	0.051 (0.003)^d^	0.023 (0.001)^d^	0.025 (0.003)^d^	0.037 (0.001)^d^
Time 1: Between Policy 1 and Uniform Ceiling Prices for Generics (Policy 2) (2010.10~2012.3)	-0.020 (0.005)^d^	-0.019 (0.007)^b^	-0.039 (0.008)^d^	-0.011 (0.001)^d^	-0.014 (0.004)^c^	-0.020 (0.002)^d^
Time 2: Between Policy 2 and Pay-for Performance Program (Policy 3) (2012.4~2013.6)	0.013 (0.004)^b^	0.004 (0.009)	0.040 (0.010)^c^	-0.007 (0.002)^d^	-0.014 (0.005)^b^	-0.018 (0.003)^d^
Time 3: Between Policy 3 and Extended Incentive Program (Policy 4) (2013.7-2014.6)	0.011^a^ (0.006)	0.015^a^ (0.012)	-0.018 (0.013)	0.001 (0.002)	0.004 (0.007)	-0.010 (0.003)^b^
Time 4: After Policy 4 (2014.7~2017.12)	0.014 (0.005)^a^	0.000 (0.010)	0.038 (0.011)^c^	0.002 (0.002)	0.008 (0.005)	0.014 (0.001)^d^
Policy 1 (2010. 10)	0.222 (0.050)^d^	-0.095 (0.079)	0.094 (0.087)	-0.018 (0.015)	-0.130 (0.062)^a^	-0.174 (0.024)^d^
Policy 2 (2014. 4)	-0.858 (0.041)^d^	-1.241 (0.081)^d^	-2.155 (0.094)^d^	-0.636 (0.017)^d^	-0.880 (0.041)^d^	-0.917 (0.025)^d^
Policy 3 (2013.7)	0.022 (0.044)	-0.008 (0.085)	-0.154 (0.093)	0.012 (0.017)	0.084 (0.052)	0.046 (0.023)^a^
Policy 4 (2014.7)	0.010 (0.052)	-0.024 (0.080)	-0.164 (0.090)	0.013 (0.016)	0.109 (0.042)^b^	0.063 (0.022)^b^
R^2 e^	0.2422	0.1977	0.4492	0.3046	0.1076	0.4512
F (p)^f^	25.04	19.59	67.03	36.15	10.56	69.61
D^g^	1.44	1.47	1.49	1.65	1.28	1.46

a^a^*P *< .1, b^b^*P *< .05, c^c^*P *< .01, d^d^*P *< .001, ^e^R-squared (fit statistics); ^f^ F test, ^g^ Durbin-watson statistic. During the study period (January 1, 2001 – December 31, 2017), US$ 1 was equivalent to KRW 1131.84, on average.

 For otitis media, there were immediate drops in monthly pharmaceutical expenditures of KRW 1241, on average, after Uniform Ceiling Prices for Generics (Policy 2). However, the monthly drug expenditures increased by KRW 15 between the Pay-for-Performance Program (Policy 3) and the Extended Incentive Program (Policy 4). For urinary tract infections, the level of monthly total drug expenditures immediately dropped after the Uniform Ceiling Prices for Generics policy (Policy 2) (by KRW 2155). It gradually increased by KRW 40 per month between the Uniform Ceiling Prices for Generics policy (Policy 2) and Pay-for-Performance Program (Policy 3) and KRW 38 per month after the Extended Incentive Program (Policy 4) (Table 2, left panel).

 Estimated results for antibiotics showed similar patterns, with the magnitude of the estimates smaller than the corresponding estimates for monthly total drug expenditure. The level of antibiotics spending dropped immediately after the Outpatient Prescription Incentive Program (Policy 1), by KRW 130 and 174 for otitis media and urinary tract infections, respectively. Immediate drops were also estimated after the Uniform Ceiling Prices for Generics (Policy 2) by KRW 636, 880, and 917 for upper respiratory infections, otitis media, and urinary tract infections, respectively.

 There were monthly increases in antibiotic expenditures by KRW 23 (upper respiratory infection), KRW 25 (otitis media), and KRW 37 (urinary tract infection) per month before the Outpatient Prescription Incentive Program (Policy 1). A decreasing trend by KRW 11 (upper respiratory infections), KRW 14 (otitis media), and KRW 20 (urinary tract infections) was noted between Policy 1 and the Uniform Ceiling Prices for Generics (Policy 2). We estimated continued drops in monthly expenditure trends between Policy 2 and the Pay-for-Performance Program (Policy 3) by KRW 7, 14, and 18 for upper respiratory infections, otitis media, and urinary tract infections, respectively. However, the pattern eventually turned upward after the Extended Incentive Program (Policy 4). This reversal was significant only for urinary tract infections (by KRW 14) (Table 2, right panel).

 As shown in Table 3, we re-estimated the policy impact on monthly total drug expenditures using quantile regressions. For upper respiratory infections, the monthly drug expenditures increased more in the upper quantiles of the conditional distribution of drug expenditures in all time segments; eg, by KRW 6 in the 10th quantile versus KRW 39 in the 90th quantile before the Outpatient Prescription Incentive Program (Policy 1) and by KRW 12 in the 10th quantile versus KRW 23 at the 90th quantile after the Extended Incentive Program (Policy 4). These results imply that high-spending clinics drove the increase. The incremental drop in the level of pharmaceutical expenditures after the Uniform Ceiling Prices for Generics (Policy 2) was also larger in the upper quantiles (eg, by KRW 349 at the 10th versus by KRW 1376 at the 90th) (top panel, Table 3).

**Table 3:** Effects of Pharmaceutical Policies on Pharmaceutical Expenditure by Subgroup: Results of a Quantile Regression

**Key Independent Variables and Intercepts**	**Regression Estimate (Standard Error) **				
	**Quantile Group by Monthly Total Pharmaceutical Expenditure Per Prescription (in 1000 KRW)**				
	**0.1**	**0.25**	**0.5**	**0.75**	**0.9**
**Upper Respiratory Infection**					
Intercept	1.707 (0.020)^d^	3.370 (0.017)^d^	5.393 (0.017)^d^	8.368 (0.026)^d^	12.977 (0.053)^d^
Time 0: Before the Outpatient Prescription Incentive Program (Policy 1) (2008.1~2010.9)	0.006 (0.001)^d^	0.012 (0.001)^d^	0.019 (0.001)^d^	0.028 (0.001)^d^	0.039 (0.002)^d^
Time 1: Between Policy 1 and Uniform Ceiling Prices for Generics (Policy 2) (2010.10~2012.3)	-0.006 (0.002)^c^	-0.014 (0.002)^d^	-0.020 (0.002)^d^	-0.028 (0.003)^d^	-0.023 (0.004)^d^
Time 2: Between Policy 2 and Pay-for Performance Program (Policy 3) (2012.4~2013.6)	0.009 (0.003)^c^	0.014 (0.002)^d^	0.016 (0.003)^d^	0.019 (0.004)^d^	0.003 (0.007)
Time 3: Between Policy 3 and Extended Incentive Program (Policy 4) (2013.7-2014.6)	0.012 (0.003)^c^	0.016 (0.003)^d^	0.018 (0.004)^d^	0.016 (0.005)^b^	0.020 (0.010)^a^
After Policy 4 (2014.7~2017.12)	-0.010 (0.003)^c^	-0.010 (0.003)^c^	-0.007 (0.003)^a^	0.006 (0.005)	0.023 (0.009)^b^
Policy 1 (2010.10)	0.101 (0.022)^d^	0.117 (0.018)^d^	0.158 (0.020)^d^	0.153 (0.032)^d^	0.084 (0.050)
Policy 2 (2014.4)	-0.349 (0.024)^d^	-0.499 (0.022)^d^	-0.725 (0.026)^d^	-0.955 (0.036)^d^	-1.376 (0.067)^d^
Policy 3 (2013.7)	-0.094 (0.028)^c^	-0.098 (0.024)^d^	-0.097 (0.026)^c^	-0.046 (0.038)	0.095 (0.082)
Policy 4 (2014.7)	-0.038 (0.022)	-0.064 (0.022)^b^	-0.037 (0.026)	-0.030 (0.036)	-0.106 (0.076)
**Otitis Media**					
Intercept	0.621 (0.039)^d^	3.999 (0.038)^d^	5.768 (0.034)^d^	8.310 (0.062)^d^	14.722 (0.204)^d^
Time 0: Before the Outpatient Prescription Incentive Program (Policy 1) (2008.1~2010.9)	0.013 (0.001)^d^	0.017 (0.001)^d^	0.024 (0.001)^d^	0.034 (0.001)^d^	0.040 (0.002)^d^
Time 1: Between Policy 1 and Uniform Ceiling Prices for Generics (Policy 2) (2010.10~2012.3)	-0.011 (0.002)^d^	-0.019 (0.002)^d^	-0.028 (0.002)^d^	-0.035 (0.003)^d^	-0.037 (0.006)^d^
Time 2: Between Policy 2 and Pay-for Performance Program (Policy 3) (2012.4~2013.6)	-0.003 (0.003)	0.003 (0.003)	0.003 (0.003)	0.006 (0.005)	0.005 (0.008)
Time 3: Between Policy 3 and Extended Incentive Program (Policy 4) (2013.7-2014.6)	0.011 (0.004)^a^	0.006 (0.004)	0.007 (0.004)	0.004 (0.007)	0.006 (0.012)
Time 4: After Policy 4 (2014.7~2017.12)	0.000 (0.004)	0.006 (0.003)	0.010 (0.003)^b^	0.017 (0.006)^b^	0.022 (0.010)^a^
Policy 1 (2010.10)	0.047 (0.027)	0.090 (0.020)^d^	0.077 (0.002)^c^	0.024 (0.037)	-0.010 (0.064)
Policy 2 (2014.4)	-0.665 (0.028)^d^	-0.840 (0.023)^d^	-1.033 (0.027) ^d^	-1.320 (0.038)^d^	-1.685 (0.075)^d^
Policy 3 (2013.7)	0.032 (0.032)	0.058 (0.028)^a^	0.131 (0.030)^d^	0.125 (0.045)^b^	0.152 (0.088)
Policy 4 (2014.7)	-0.027 (0.026)	0.044 (0.024)	0.112 (0.030)^c^	0.128 (0.044)^b^	0.140 (0.089)
**Urinary Tract Infection**					
Intercept	3.116 (0.033)^d^	4.940 (0.028)^d^	7.416 (0.038)^d^	10.963 (0.066)^d^	17.478 (0.150)^d^
Time 0: Before the Outpatient Prescription Incentive Program (Policy 1) (2008.1~2010.9)	0.017 (0.001)^d^	0.025 (0.001)^d^	0.039 (0.002)^d^	0.058 (0.003)^d^	0.091 (0.006)^d^
Time 1: Between Policy 1 and Uniform Ceiling Prices for Generics (Policy 2) (2010.10~2012.3)	-0.004 (0.003)	-0.012 (0.003)^c^	-0.021 (0.004)^d^	-0.046 (0.007)^d^	-0.050 (0.018)^b^
Time 2: Between Policy 2 and Pay-for Performance Program (Policy 3) (2012.4~2013.6)	-0.002 (0.005)	0.007 (0.005)	0.009 (0.007)	0.052 (0.011)^d^	0.077 (0.027)^b^
Time 3: Between Policy 3 and Extended Incentive Program (Policy 4) (2013.7-2014.6)	-0.011 (0.006)	-0.020 (0.006)^b^	-0.017 (0.008)^a^	-0.023 (0.014)	-0.024 (0.037)
Time 4: After Policy 4 (2014.7~2017.12)	0.015 (0.015)^b^	0.023 (0.005)^d^	0.033 (0.007)^d^	0.040 (0.012)^b^	0.054 (0.029)
Policy 1 (2010.10)	-0.075 (0.039)	-0.036 (0.038)	-0.016 (0.056)^d^	0.126 (0.088)	0.165 (0.194)
Policy 2 (2014.4)	-0.694 (0.048)^d^	-1.093 (0.045)^d^	-1.631 (0.056)^d^	-2.389 (0.098)^d^	-3.850 (0.239)^d^
Policy 3 (2013.7)	0.090 (0.051)	0.017 (0.045)	-0.024 (0.066)	-0.349 (0.105)^c^	-0.563 (0.245)^a^
Policy 4 (2014.7)	0.049 (0.042)	0.092 (0.042)^a^	-0.001 (0.058)	-0.254 (0.101)^a^	-0.662 (0.230)^b^

a^a^*P *< .1, b^b^*P *< .05, c^c^*P *< .01, d^d^*P *< .001. During the study period (January 1, 2001 – December 31, 2017), US$ 1 was equivalent to KRW 1131.84, on average.

 Similarly, we estimated a more significant immediate drop and a bigger monthly increase in total drug expenditures in the upper quantiles for otitis media. There was a monthly increase in total drug expenditures from KRW 13 (at the 10th quantile) to KRW 40 (at the 90th quantile) before the Outpatient Prescription Incentive Program (Policy 1). These increases became statistically insignificant or were attenuated slightly after the Extended Incentive Program (Policy 4). After the Uniform Ceiling Prices for Generics (Policy 2), the monthly total drug expenditures declined by KRW 665 at the 10th quantile, compared to KRW 1685 at the 90th quantiles (middle panel, Table 3).

 For urinary tract infections, the estimates were higher than the other two target diseases, given that the unadjusted drug expenditure was higher for this disease than others. The monthly total drug expenditure trended upward in all segments. The extent of the increase was greater in the upper quantiles, with the difference between the 10th quantile and the 90th quantile almost five to tenfold. For example, the expenditure increased by KRW 17 at the 10th quantile and KRW 91 at the 90th quantile before the Outpatient Prescription Incentive Program (Policy 1). The corresponding estimates were KRW 15 and KRW 54 after the Extended Incentive Program (Policy 4). The decrease after the universal price cut by the Uniform Ceiling Prices for Generics (Policy 2) was KRW 694 at the 10th quantile versus KRW 3850 at the 90th quantile, and by KRW 662 at the 90th quantile after the Extended Incentive Program (Policy 4) (bottom panel, Table 3).

## Discussion

 This study assesses the overall effects of four policies targeting pharmaceutical expenditure containment via direct price cuts or inducing changes in physicians’ prescription behaviors. Before introducing the policies, total drug and antibiotic spending increased in all three target diseases during the baseline period. Estimation results showed that the various incentive programs had minimal to insignificant effects on curbing expenditure per prescription. The pharmaceutical expenditure per prescription tended to reduce immediately after the universal price cut policy; however, the cost trend increased afterward. The increase in total drug costs per prescription grew higher after each policy was sequentially introduced. In contrast, antibiotic spending per prescription remained at a lower level than before the policies.

 We used quantile regression to estimate the heterogeneous policy effects by the clinics’ claim volumes. Our analysis showed that the policy effect on cost containment was largest for high-volume clinics. We also found that these high-volume clinics had the fastest growth in total expenditure across the study period. The current incentive structure provides the same incentives to all clinics regardless of their relative prescription volume.^22^ Differentiating the incentive formula by claims volume would maximize the policy effect by guiding the higher spending clinics to respond more sensitively to the incentive.

 Some studies have shown that financial incentives influence prescription behavior.^23,24^ For example, Burkhard et al^25^ showed that doctors reacted to changes in reimbursement schemes in Switzerland by adjusting prescription volumes. Liu et al^26^ estimated that physicians are likely to increase their prescription volume to cover their financial losses from drug price reductions using claims data in Taiwan. This may be specific to Taiwan, where physicians both prescribe and dispense drugs.^27,28^ Jacobson et al^28^ focused on the Medicare Prescription Drug, Improvement, and Modernization Act in the United States, which reduced Medicare payment rates for outpatient chemotherapy drugs in 2003. They showed that physicians switched drugs with the largest cuts in profitability to other high-margin drugs.^22,29^

 We anticipate that physicians work for the welfare of their patients as a perfect agent. However, a physician may behave as an imperfect agent in the real world due to asymmetric information.^30^ Prescriptions are a complex process affected by a physician, patient, policy, and environmental factors.^10,31,32-35^ One investigation^36^ reviewed 33 related studies for determinants of prescribing behavior and found identifying physicians, drug price, and marketing factors as the frequently cited determinants. A recent survey of Iranian physicians^37^ reported that environmental and marketing factors did not have an influence, whereas drug characteristics, patient conditions, and insurance type affect doctors’ prescription decisions. Hellerstein^38^ found that doctors in Health Maintenance Organizations or pre-paid insurance schemes were more likely to prescribe cheaper generic drugs.

 The Pay-for-Performance Program reimburses physicians for their prescription behaviors and efficiency.^22,39^ If doctors react to financial inducements, we expect that an incentive scheme such as the Outpatient Prescription Incentive Program in Korea may affect physicians’ prescription behaviors and consequently contribute to the suppression of drug cost. However, our findings showed that it was insufficient to induce the appropriate use of drugs similar to previous studies.^40-42^ Although, the Pay-for-Performance System that dis-incentivizes clinics that overuse antibiotics could partially suppress antibiotic use, the increases in total drug expenditure per prescription were more significant in our study.^43,44^

 A price cut may directly contribute to the reduction of pharmaceutical spending in the short run.^4,45-48^ However, physicians or patients may change their behaviors in response to the price cut in the long term, yielding unexpected results in drug spending.^49^ Carone et al^6^ mentioned that price cuts might achieve cost containment in the short run, but increasing the drug volume prescribed over time might counterbalance these effects. Chu et al^7^ observed physicians’ behavior in increasing prescription duration and the number of drugs per prescription compared to the reduction of drug reimbursement rates in Taiwan. Hsu et al^8^ also showed that Taiwanese physicians shifted their drug choice from targeted to non-targeted drugs after reductions in reimbursements.

 The present study provides real-world evidence of the impact of pharmaceutical cost-containment policies targeting the most frequent conditions encountered in outpatient settings. Its study population encompassed all operating clinics in the capital city of Seoul and six other metropolitan cities, in which approximately half of the Korean population resided as of February 2019. We also identified the heterogeneous response of the clinics to various pharmaceutical cost containment measures by the conditional magnitude of claims (ie, business scales).

 We assessed the medium-term effect of those policies by utilizing 10 years of claims data. Han et al^4^ similarly analyzed antibiotic prescription behavior for patients with acute upper respiratory tract infections, acute lower respiratory tract infections, and otitis media. They obtained mixed results for the Outpatient Prescription Incentive Program in October of 2010. They found that the Uniform Ceiling Prices for Generics lowered drug spending immediately through the price effect, but this effect later diminished. Han et al^4^ only observed eight months after the Uniform Ceiling Prices for Generics in April of 2012 and used a limited clinic sample. Park and Han^5^ also reported that outpatient incentive policies had a limited effect on pharmaceutical spending. Here, we shed light on the longer-term impacts of relevant policies.

 The underlying mechanism of physicians’ responses remains unidentified in the present study. Exploiting a vast amount of real-world data from all clinics assures our results’ external validity. This generated a tradeoff for aggregating the data monthly without micro-level analyses delineating at the individual claim level. One critical spill-over effect of policies to contain pharmaceutical prices would be a potential negative impact on the quality of care. A recent systematic review revealed that not embedding the pay-for-performance system with specific outcome objectives can have mixed effects on patient health outcomes.^50,51^ We acknowledge that no control group exists in our research context, since Korea has a single public health insurer and all Koreans and legal residents are beneficiaries. The policies of interest impact all providers. There has been only limited empirical investigation of the impact of those policies on patient outcomes,^49^ and most relevant research has focused on the significance of cost-sharing rather than policies to modulate drug costs.^52-55^ Future studies must include patient health outcomes.

## Conclusion

 Enforcement of cost-containment policies, especially price reduction, had an immediate effect on reducing pharmaceutical expenditure, but there was no reversal in the upward trend afterward. Medical providers may find ways to circumvent cost-containment, requiring the need for well-organized and effective incentive schemes. Our findings of a more significant increase in pharmaceutical expenditures among high-volume clinics imply that policy measures could target specifically high-spending clinics.

## Acknowledgments

 Research support from the Korea Institute of Public Finance is gratefully acknowledged. The content is solely the responsibility of the authors and does not necessarily represent the official view of the Korea Institute of Public Finance. The Korea Institute of Public Finance was not involved in the preparation and submission of this manuscript.

## Ethical issues

 The Public Institutional Review Board waived study review for human research (P01-201902-22-002).

## Competing interests

 WHK was an employee of the Korea Institute of Public Finance when this research was done. Other authors declare that they have no competing interests.

## Authors’ contributions

 WHK and EH conceived of this study. HJK performed data collecting, cleaning, and analysis, and EH verified the analytical methods. HJL and WHK performed a literature review on related studies and policies. WHK, HJL, and EH drafted the manuscript. All authors discussed the analytical methods, the results presented, and the completed manuscript.

## Availability of data and material

 Data will be made available on responsible request. The datasets analyzed during the current study are not publicly available.

## Funding

 This research concerns aspects of a research project of the Korea Institute of Public Finance which is a public organization. The study was supported by the Korea Institute of Public Finance.
